# Cognitive and Metacognitive Mechanisms of Change in Metacognitive Training for Depression

**DOI:** 10.1038/s41598-017-03626-8

**Published:** 2017-06-14

**Authors:** Lena Jelinek, Niels Van Quaquebeke, Steffen Moritz

**Affiliations:** 1 University Medical Center Hamburg-Eppendorf, Department of Psychiatry and Psychotherapy, Martinistr. 52, 20246 Hamburg, Germany; 2 Kühne Logistics University, Großer Grasbrook 17, 20457 Hamburg, Germany

## Abstract

Metacognitive Training for Depression (D-MCT), a low-threshold group intervention, has been shown to improve depressive symptoms. It aims at the reduction of depression by changing dysfunctional cognitive as well as metacognitive beliefs. The purpose of the present study was to investigate whether the mechanisms of change in D-MCT are cognitive (and thus primarily concern the content of cognition) or metacognitive in nature. Eighty-four outpatients with depression were included in a randomized controlled trial comparing D-MCT to an active control intervention. Level of depression, dysfunctional cognitive beliefs (DAS), and metacognitive beliefs (MCQ subscales: Positive Beliefs, Negative Beliefs, Need for Control) were assessed before (t0) and after treatment (t1). Severity of depression was also assessed 6 months later (t2). Linear regression analyses were used to determine whether change in depression from t0 to t2 was mediated by change in cognitive vs. metacognitive beliefs from t0 to t1. D-MCT’s effect on change in depression was mediated by a decrease in dysfunctional metacognitive beliefs, particularly ‘need for control’. Our findings underline that one of the key mechanisms of improvement in D-MCT is the change in metacognitive beliefs. The current study provides further support for the importance of metacognition in the treatment of depression.

## Introduction

Besides the psychological and somatic symptoms that represent core diagnostic criteria of affective disorders, depression is characterized by maladaptive cognitive beliefs and biases, which lie at the heart of cognitive behavioral theories of depression^1^. Although dysfunctional beliefs have been shown to be relatively stable^2^, they do appear to be malleable and, in consequence, represent a major target of cognitive behavioral therapy (CBT). As a result, research until now has converged on the notion that depressive symptoms can be reduced via the modification of cognitive biases^3, 4^. However, the results are not fully conclusive^3^. For instance, it is unclear which mechanisms of change are evoked by different (cognitive) treatments for depression. Identification of the specific process variables that mediate patient outcomes could help to improve our understanding of how psychotherapy works and, as a result, initiate specific refinements.

Over the past decades, several treatment approaches with a metacognitive focus have been developed in parallel, including Metacognitive Therapy in Great Britain^5^ and Metacognitive Training in Germany (the latter was originally developed for psychosis^6^ and has been shown effective in a recent meta-analsis^7^). Although both approaches use the word “metacognition” in their name, they are quite distinct therapeutic interventions. Metacognitive Therapy developed by Wells represents a generic approach for various psychological disorders that considers working on specific cognitive content irrelevant. In contrast, Metacognitive Training developed by Moritz targets disorder-specific cognitive biases (e.g., jumping to conclusions in psychosis) by addressing general mechanisms of thinking (the metacognitive perspective) as well as the content of dysfunctional thoughts (the cognitive perspective). In other words, Moritz’s approach to Metacognitive Training differs from Wells’ approach to Metacognitive Therapy in that it targets both the content of cognition as well as metacognitive beliefs, whereas Wells’ approach is concerned with meta-level cognition and does not address the specific content of intrusive thoughts. Moritz’s Metacognitive Training is thus fully compatible with a general CBT treatment approach. It adopts both the cognitive and metacognitive perspectives, which are not deemed exclusive but rather complementary. As mentioned, Moritz’s approach was originally developed for patients with psychosis. Over the past decade, he and his colleagues have developed specific metacognitive training programs for depression, obsessive compulsive disorder, and borderline personality disorder, which in their general structure and use of exercises were inspired by Metacognitive Training for psychosis^6^. As a result, all these trainings attempt to challenge disorder-specific cognitive biases through the use of creative and engaging exercises supported by a multimedia presentation.

**Figure 1:**
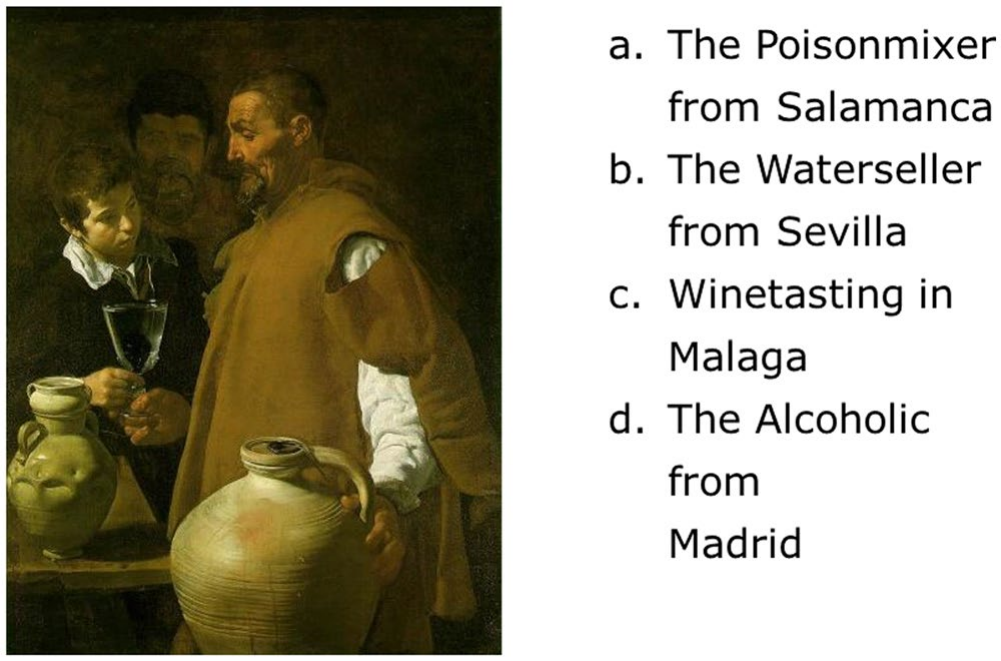
Exercise in D-MCT module 7 on “Jumping to Conclusions: Mind reading”. (To modify their dysfunctional thought pattern and to demonstrate that mind reading is not possible, patients are asked to guess the title of the painting and “read the mind” of the artist). The correct answer is “b” (“The Waterseller from Sevilla” by Diego Velázquez, 1623).

The present study used Metacognitive Training for Depression (D-MCT)^8^, which uses content tailored to the specific problems of individuals with depression. It seeks to enable group members to recognize and correct the often automatic and unconscious thought patterns that accompany depression. In addition to the depressive thought patterns that are also targeted in CBT (e.g., overgeneralization), general cognitive biases that have been identified by basic cognitive research (e.g., mood-congruent memory) are also addressed. Finally, dysfunctional coping strategies (i.e., thought suppression, rumination) are targeted, which are also addressed in the Metacognitive Therapy developed by Wells. D-MCT thus uses established techniques of CBT but complements them with new, engaging exercises (for an example, see Fig. 1) as well as a broader metacognitive perspective. D-MCT has been developed as a low-threshold (i.e., minimal demands) group concept in that patients are not required to talk about their individual problems but may if they wish; either way, they still experience how cognitive biases work and influence one’s mood. Moreover, the threshold is low with regard to administration; the preparation time is short because the therapy sessions are highly standardized (through the use of multimedia slide based presentations) and are thus easy to administer. Moreover, new patients may join the group in any session as the training is delivered in an open group format.

We recently reported the efficacy of D-MCT compared with an active control intervention (health training) in a randomized controlled trial (RCT) regarding depression, dysfunctional beliefs, and quality of life with medium (to large) effect sizes^9, 10^, but little is known about the mechanisms of change. We expect that, in line with general principles of CBT, patients benefit through the modification of their cognitive beliefs (i.e., the content of their cognition) as well as by reducing their dysfunctional metacognitive beliefs. It has been shown that depression is associated with dysfunctional metacognitive beliefs^11, 12^. For D-MCT, we were able to show in an initial study that over the course of the training, dysfunctional cognitive and metacognitive beliefs were reduced at medium effect^9^. However, due to the study’s design (uncontrolled pilot study, only two assessments), mechanisms of change could not be investigated, and it remains unclear whether change in beliefs represents a cause or a consequence of the improvement in depression. In our recent trial^10^, we also reported a larger decrease in dysfunctional cognitive beliefs in the D-MCT compared to a control group over time; change in metacognitive beliefs, however, were not reported, and mechanisms of change were not investigated.

The aim of the present study is thus to extend our current understanding by providing a more comprehensive picture. To that end, we analyzed data on change in metacognitive beliefs and reanalyzed previous data from our recent RCT on the effectiveness of D-MCT^10^ with regard to mechanisms of change. In our effort to develop a more complete understanding, we hypothesized a larger reduction of metacognitive beliefs in the D-MCT compared to the control group. Moreover, in line with cognitive behavioral models of depression^1^, which assume that maladaptive cognitive beliefs are involved in the development and maintenance of depressive symptoms^3, 4^, we expected that a better outcome 6 months after D-MCT treatment would be mediated by an improvement in dysfunctional cognitions. To this end, and to conclusively address the specific cognitions that the D-MCT training affects, we explored treatment effects over the course of treatment on two types of dysfunctional cognitions: cognitive beliefs (i.e., the content of cognitions as measured by the Dysfunctional Attitude Scale) and metacognitive beliefs (as assessed by the Metacognition Questionnaire).

## Methods

### Design

Design of the study has been described elsewhere^10^. We performed a parallel RCT comparing two group interventions, which were administered in addition to an intensive psychosomatic outpatient treatment program: D-MCT (experimental group) and health training (HT, active control group). Participants were allocated to D-MCT or HT after baseline assessment according to a fixed computer-generated randomization plan with a 1:1 allocation ratio. Patients met with research staff blind to treatment allocation at baseline (t0) as well as 4 weeks (post-treatment, t1) and 6 months later (follow-up, t2) for a face-to-face assessment. To keep raters blind with respect to group allocation (to prevent the Rosenthal effect), patients were not assessed at the same time at which groups met and were instructed (and repeatedly reminded) not to disclose group assignment to the raters. All participants gave written informed consent prior to participation. The research was carried out in accordance with approved guidelines and in accordance with the Declaration of Helsinki and its amendments. The study protocol was approved by the Ethics Committee of the German Psychological Association and was registered at the German Clinical Trials Register (#DRKS00007907, registration date: 2015/04/30).

### Participants

Eighty-four patients (62 women and 22 men; age: *M* = 45.5, *SD* = 9.89; years of formal school education: *M* = 10.61, *SD* = 1.69; number of major depressive episodes (MDE): *M* = 3.03, *SD* = 5.69) with a depressive disorder treated at a psychosomatic outpatient day clinic were included. Diagnosis of a current MDE, recurrent depression, or dysthymia was verified using the Mini International Neuropsychiatric Interview (MINI)^13^. The MINI was slightly modified in order to differentiate recurrent vs. single-episode major depressive disorder as well as double depression. At baseline, 36 patients (43%) were diagnosed with a single MDE, 47 (56%) with recurrent depression, and one with dysthymia (1%). At intake, more than half of the patients (*n* = 43) met criteria for at least one comorbid disorder, according to the MINI. Demographic data is presented in Table 1. Patients were excluded if they were younger than 18 or older than 65 years of age, did not fulfill criteria for a current or recurrent MDE or dysthymia, suffered from psychotic symptoms (i.e., hallucinations, delusions, or mania), were currently suicidal, or showed signs of intellectual disability (estimated IQ < 70). Use of psychotropic medications was tolerated. Completion rate at t1 was 94.0% and at t2 was 71.4%. Participants who completed the 6-month follow-up did not significantly differ from patients who were lost to follow-up on baseline demographic, psychopathological, or cognitive characteristics.

**Table 1:** Demographics: Frequencies, Means, and Standard Deviations (in Brackets).

Variable	D-MCT (*n* = 41)	HT (*n* = 43)	Statistics (D-MCT vs. HT)
Age (years)	44.05 (10.23)	46.81 (9.27)	*t*(82) = 1.30, *p* = 0.198
Gender (female/male)	29/12	33/10	χ^2^(1) = 0.39, *p* = 0.531
Years of formal school education	10.61 (1.48)	10.62 (1.88)	*t*(82) = 0.02, *p* = 0.986
Verbal intelligence (T-Score)	52.20 (8.64)	52.26 (8.80)	*t*(77) = 0.03, *p* = 0.977
Job status (working/sick leave/unemployed)	2/25/14	2/22/19	χ^2^(2) = 0.90, *p* = 0.637
Alcohol consumption per week (in g)	18.55 (34.33)	22.96 (37.44)	*t*(79) = 0.55, *p* = 0.582
Number of MDE (including present)	4.11 (7.79)	2.00 (2.09)	*t*(38.64) = 1.55, *p* = 0.128
Medication			Cramer-V = 0.15, *p* = 0.582
antidepressant	27	27	
antipsychotic	0	2	
combination	1	1	
none	13	13	

*Note* MDE = major depressive episode.

#### Treatment Conditions

All patients received a standard intensive psychosomatic outpatient treatment for 5 days a week, 8 hours a day. The program consisted of individual therapy sessions, group therapies (e.g., social skills training), occupational therapies, physical training, weekly ward rounds, etc. An extensive D-MCT treatment manual is available in German^8^; material in other languages, including English, can be downloaded at www.uke.de/depression. The HT group was equivalent to D-MCT in terms of the frequency (eight sessions, two sessions per week), duration of sessions (60 min), and group format. For HT, the weekly format consisted of one group walking session (60 min) and one psychoeducation session on health topics such as stress reduction or nutrition (60 min).

### Outcome measures

Two outcomes were investigated^10^. The primary outcome measure was the total score of the Hamilton Depression Rating Scale (HDRS, 17-item version)^14^. The secondary outcome measure was self-assessed depression as measured by the well-established Beck Depression Inventory (BDI-I)^15^.

### Potential cognitive mediators

- The Dysfunctional Attitudes Scale (DAS)^16^ was used to assess dysfunctional cognitive beliefs (i.e., the content of cognition). The DAS includes 40 items that are rated by participants on a 7-point Likert scale. Reliability (internal consistency ranges between α = 0.88 and 0.94) and validity (correlations with other measures of depression range between *r* = 0.71 and 0.85) have been documented. In the current study, internal consistency was excellent, with α = 0.93.
- To assess metacognitive beliefs, the following three subscales of the Metacognitions Questionnaire 30 (MCQ-30)^17^ were used: (1) Positive Beliefs (PB) about worry (e.g., “Worrying helps me to get things sorted in my mind”, 6 items); (2) Negative Beliefs (NB) about thoughts concerning uncontrollability and danger (e.g., “When I start worrying I cannot stop”, 6 items), and (3) beliefs about the need to control thoughts (Need for Control [NFC], e.g., “Not being able to control my thoughts is a sign of weakness”, 6 items). Only these three subscales were administered as they are most related to depression^12^ and the two remaining MCQ subscales, “cognitive confidence” and “cognitive self-consciousness”, were less affected by D-MCT in the pilot study^9^. The MCQ-30 showed good psychometric qualities, with a test-retest reliability of *r* = 0.75 and internal consistencies ranging from α = 0.72–0.93 for the subscales and 0.93 for the total score in previous studies. In the current study, internal consistencies were acceptable, with α = 0.75 for PB, α = 0.78 for NB, and α = 0.71 for NFC.

### Strategy of data analysis

IBM SPSS 22.0 software was used for all analyses. All randomized patients were included in the analyses (intention to treat), and missing outcome data were imputed by the expectation-maximization (EM) algorithm trimmed to fall between the minimum and maximum of possible values. For mediation analysis, D-MCT treatment was coded as 1 and HT treatment as 0. A treatment effect in the analyses thus refers to effects of D-MCT above and beyond HT. To capture the change in depression (HDRS and BDI), standardized residualized change scores using a simple linear regression model in which baseline scores predicted follow-up scores (t0 to t2) were calculated. To capture change in the mediators (DAS and MCQ subscales), standardized residualized change scores were calculated in which baseline scores predicted post-assessment scores (t0 to t1). The subsequent mediation analysis thus determines to what extent the change in depression from t0 to t2, i.e., from baseline to 6-months follow-up, that was brought about by D-MCT above and beyond HT can be explained by change in the mediator variables in the treatment period, i.e., from baseline to end of treatment 4 weeks later (t0–t1). The mediational analyses met all of the criteria for the definition and evaluation of mediators within RCTs as established by Kraemer and colleagues^18^.

The analysis was conducted using an SPSS macro PROCESS developed by Hayes (version v2.13 downloaded 3 March 2015)^19^. The analysis allows delineating the effects of each of the proposed mediators separately while controlling for the others^20^. It thus presents a rather conservative test for estimating individual mediation effects. To correct for potential biases of non-normality in the sample, we bootstrapped results 1,000 times. When the effect range (LL = lower limit to UL = upper limit) of the 95% confidence interval (CI) does not include zero, the null hypothesis is considered rejected. When the 95% CI does include zero, the mediation hypothesis is considered rejected. Following Preacher and Kelley^21^, we additionally computed κ^2^ for significant mediators as effect size. Statistically, this is currently only possible without the consideration of the simultaneous effects of other covariates (i.e., mediators). Respective effect sizes can be interpreted in analogy to Cohen’s *r* ^2^, that is, 0.01, 0.09, and 0.25 constitute small, medium, and large effects, respectively. Effect sizes for ANOVAs are reported according to Kinnear and Gray^22^ with η_p_^2^ ≈ 0.01, η_p_^2^ ≈ 0.06, and η_p_^2^ ≈ 0.14, corresponding to small, medium, and large effects, respectively.

## Results

Groups were similar on psychopathological and sociodemographic data as well as medication (see Table 1). Mean scores of the three MCQ subscales are presented in Table 2 (for DAS results, see Jelinek^10^), showing greater reduction on all three subscales in the D-MCT group in comparison to the HT group at a medium to large effect size (η_p_^2^ = 0.048 to 0.101). Zero-order correlations between all involved variables over the different time points (t0, t1, t2) are displayed in Table 3. Correlations were highest within the same constructs over time (e.g., DAS t0 with DAS t1) but, as expected, the cognitive (DAS) and metacognitive (MCQ) scales were also significantly correlated ^cf^.,^12^.

**Table 2:** Group Comparisons (Intention to Treat Sample) and Results of the ANCOVAs for Metacognitive Beliefs for Pre- and Post-Assessment.

	Pre-Assessment (t0)			Post-Assessment (t1)		
	D-MCT (*n* = 41)	HT (*n* = 43)	*t* test	D-MCT (*n* = 41)	HT (*n* = 43)	ANCOVA^a^
MCQ (PB)	13.23 (4.16)	12.76 (3.64)	*t*(82) = 0.558, *p* = 0.578	11.43 (2.85)	13.05 (3.51)	*F*(1, 81) = 12.041, *p* = 0.001, η_p_^2^ = 0.129
MCQ (NB)	18.51 (3.98)	17.24 (4.01)	*t*(82) = 1.465, *p* = 0.147	16.00 (3.93)	17.28 (4.02)	*F*(1, 81) = 8.327, *p* = 0.005, η_p_^2^ = 0.093
MCQ (NFC)	14.12 (4.66)	13.43 (3.26)	*t*(71.28) = 0.791, *p* = 0.432	11.94 (3.97)	13.18 (4.37)	*F*(1, 81) = 11.445, *p* = 0.012, η_p_^2^ = 0.075

*Note* D-MCT = Metacognitive Training for Depression, HT = health training, MCQ (NB) = Metacognition Questionnaire, Negative Beliefs; MCQ (NFC) = Metacognition Questionnaire, Need for Control; MCQ (PB) = Metacognition Questionnaire, Positive Beliefs. ^a^ Difference scores (baseline to post) served as dependent variables; baseline scores were entered as a covariate. Effect sizes for η_p_^2^ refer to small (η_p_^2^ ≈ 0.01), medium (η_p_^2^ ≈ 0.06), and large (η_p_^2^ ≈ 0.14) effects, respectively.

**Table 3:** Zero-Order Correlations Between Cognitive (DAS) Metacognitive Beliefs (MCQ) for Pre- and Post-Assessment.

	1.	2.	3.	4.	5.	6.	7.	8.	9.	10.	11.	12.	13.	14.
1. DAS, t0		0.273^*^	0.291^**^	0.542^**^	0.503^**^	0.327^**^	0.799^**^	0.257^*^	0.298^**^	0.426^**^	0.420^**^	0.314^**^	0.360^**^	0.188
2. MCQ (PB), t0			0.271^*^	0.322^**^	0.200	0.056	0.133	0.602^**^	0.157	0.218	−0.043	−0.116	0.185	0.157
3. MCQ (NB), t0				0.498^**^	0.311^**^	0.249^*^	0.145	0.298^**^	0.532^**^	0.349^**^	0.239^*^	0.066	0.061	0.085
4. MCQ (NFC), t0					0.391^**^	0.252^*^	0.413^**^	0.451^**^	0.407^**^	0.662^**^	0.354^**^	0.202	0.231	0.162
5. BDI, t0						0.651^**^	0.543^**^	0.250^*^	0.258^*^	0.416^**^	0.65^**^	0.435^**^	0.574^**^	0.478^**^
6. HDRS, t0							0.471^**^	0.167	0.306^**^	0.437^**^	0.611^**^	0.633^**^	0.515^**^	0.507^**^
7. DAS, t1								0.282^*^	0.441^**^	0.550^**^	0.679^**^	0.570^**^	0.601^**^	0.444^**^
8. MCQ (PB), t1									0.337^**^	0.396^**^	0.245^*^	0.096	0.417^**^	0.271^*^
9. MCQ (NB), t1										0.589^**^	0.567^**^	0.431^**^	0.363^**^	0.285^*^
10. MCQ (NFC), t1											0.653^**^	0.502^**^	0.538^**^	0.445^**^
11. BDI, t1												0.737^**^	0.761^**^	0.632^**^
12. HDRS, t1													0.649^**^	0.661^**^
13. BDI, t2														0.775^**^
14. HDRS, t2														

*Note* DAS = Dysfunctional Attitude Scale; MCQ (NB) = Metacognition Questionnaire, Negative Beliefs; MCQ (NFC) = Metacognition Questionnaire, Need for Control; MCQ (PB) = Metacognition Questionnaire, Positive Beliefs, * = *p* < 0.05, ** = *p* < 0.01 (two-tailed).

Mediation was tested with regard to clinician-assessed (HDRS) and self-assessed (BDI) depression (Table 4). The results related to both outcomes correspond: Whereas treatment with D-MCT elicited improvement on all four (sub)scales beyond HT, only the change in NFC evoked long-term changes in depression (i.e., it is the only significant mediator). For the HDRS, the indirect effect of treatment on change in depression via NFC is 0.15 (*SE* = 0.10; BootLLCI = 0.02, BootULCI = 0.44; κ^2^ = 0.09), with a remaining significant direct effect of treatment on change in depression of 0.64 (*SE* = 0.21; LLCI = 0.21, ULCI = 1.06). For the BDI, the indirect effect of treatment on change in depression via NFC is 0.22 (*SE* = 0.11; LLCI = 0.05, ULCI = 0.52; κ^2^ = 0.13), with a remaining direct effect of treatment on change in depression of 0.59 (*SE* = 0.19; LLCI = 0.21, ULCI = 0.98). For the remaining but insignificant indirect effects of the other potential mediators that were explored, please see Table 5.

**Table 4:** Mediation Analysis Part 1: Effects of Treatment on Mediators, and Effects of Mediators (Including Treatment) on Both Outcomes.

	Mediators								Outcomes			
	DAS		MCQ (PB)		MCQ (NB)		MCQ (NC)		HDRS		BDI	
	*b*	*SE*	*b*	*SE*	*b*	*SE*	*b*	*SE*	*b*	*SE*	*b*	*SE*
Treatment	0.44*	0.21	0.71***	0.20	0.60**	0.21	0.54*	0.21	0.64**	0.21	0.59**	0.19
DAS									0.14	0.12	0.04	0.11
MCQ (PB)									−0.07	0.11	0.04	0.10
MCQ (NB)									−0.02	0.12	0.06	0.11
MCQ (NFC)									0.27*	0.21	0.40**	11
*R* ^*2*^	0.05		0.13		0.09		0.07		0.26		0.40	

*Note* Treatment = 0 (health training) or 1 (Metacognitive Training for Depression); DAS = Dysfunctional Attitude Scale; MCQ (NB) = Metacognition Questionnaire, Negative Beliefs; MCQ (NFC) = Metacognition Questionnaire, Need for Control; MCQ (PB) = Metacognition Questionnaire, Positive Beliefs. Mediators and outcomes are residuals to capture change, as described in the methods section. * = *p* < 0.05, ** = *p* < 0.01, *** = *p* < 0.001 (two-tailed).

**Table 5:** Mediation Analysis Part 2: The Indirect Effects of the Potential Four Mediators on Both Outcomes Brought About by the Treatment.

	HDRS				BDI			
	Effect	*SE*	LLCI	ULCI	Effect	*SE*	LLCI	ULCI
DAS	0.06	0.11	−0.02	0.25	0.02	0.05	−0.06	0.16
MCQ (PB)	−0.05	0.07	−0.21	0.08	0.03	0.07	−0.12	0.18
MCQ (NB)	−0.01	0.07	−0.19	0.11	0.04	0.06	−0.07	0.18
MCQ (NFC)	0.15	0.11	0.02	0.44	0.22	0.11	0.05	0.18
Total	0.14	0.11	−0.08	0.38	0.30	0.13	0.07	0.61

*Note* Treatment = 0 (health training) or 1 (Metacognitive Training for Depression); DAS = Dysfunctional Attitude Scale; MCQ (NB) = Metacognition Questionnaire, Negative Beliefs; MCQ (NFC) = Metacognition Questionnaire, Need for Control; MCQ (PB) = Metacognition Questionnaire, Positive Beliefs. Mediators and outcomes are residuals to capture change, as described in the methods section. All scores based on 1,000 bootstrap samples. CI 95%.

## Discussion

Our study provides initial insight into how D-MCT exerts its effects on depression. As predicted, D-MCT elicited improvement in cognitive (i.e., content of cognition) as well as metacognitive beliefs across treatment beyond the control treatment (HT). However, only one of the potential (meta)cognitive processes, NFC, acted as a mediator between treatment and depression decline. Moreover, only partial mediation with medium effect occurred, and thus other processes seem to be involved. The lack of a mediating role of the other two meta-cognitive subscales (NB, PB) is at first surprising. Although they were significantly reduced, with at least medium effect size over treatment in the D-MCT group, they did not seem to work as mediators, which invites further consideration. Both subscales (NB, PB) are primarily concerned with “worry” in the form of perseverative negative thinking, which has been traditionally implicated in anxiety but more recently also in depression^23^. In fact, all MCQ items were first developed for aspects of anxiety. However, only change in NFC worked as a mediator, and it worked even better than change in dysfunctional cognitive beliefs as measured by the DAS, an instrument that has quite reliably indicated the association between decrease of dysfunctional beliefs and improvement of depression^24^. Although D-MCT largely overlaps with classic CBT (which mainly aims at the modification of the ‘content’ of dysfunctional thoughts), it directly addresses the modification of dysfunctional metacognitive beliefs. A core theme of all the Metacognitive Trainings developed by Moritz is that everybody’s cognitions are prone to biases (“to err is human”). This is potentially (best) picked up by the MCQ subscale NFC, which, in consequence, is the most important mediator of change.

Because we did not use all scales of the MCQ in the present study, it remains to be shown whether the MCQ scales “cognitive confidence” (CC) and “cognitive self-consciousness” (CSC) may also act as mediators. Whereas CC has been found to predict outcome in other research^25–27^ and is generally relevant to depression, items of the CC subscale of the MCQ do not capture the content of D-MCT in this regard and instead mainly relate to subjective memory problems. To be more specific, the CC scale includes items such as “I have little confidence in my memory for actions/places/words and names” as well as “My memory can mislead me at times”. In D-MCT, module #2 is primarily concerned with memory and attention in depression. However, in this module patients learn (through exercises) about the origin of memory deficits in depression (poor selective attention, for example, due to rumination) and mood-congruent recall as well as false memories. Accordingly, they are taught that memory in general does not work passively like a video camera but is prone to biases. Although we assume that cognitive confidence in general would increase as a result of D-MCT, it is unlikely to be captured by the items of the CC subscale of the MCQ and there may even be a decline in this scale (despite successful therapy). We therefore consider the CC scale inadequate for measuring the success of the D-MCT. However, this remains to be shown in future studies.

The metacognitive model by Wells conceptually argues that the relationship between metacognitive beliefs and depression is mediated by rumination. Extending this idea to our present empirical results, this could mean that NFC may underlie the use of maladaptive coping strategies (e.g., rumination). However, use of coping strategies was not measured, so it remains to be tested whether the change in metacognitive beliefs directly impacts depression at the same time as coping strategies or whether they work in sequence, i.e., a change in metacognitive beliefs impacts depression through a change in coping (including increased activation/activities or social relations). Still, the present study is the first to empirically address the cognitive mechanisms of change in D-MCT. It specifically suggests that D-MCT’s ‘active essence’ is the encouraging of patients to disengage from dysfunctional metacognitive beliefs. This may ultimately enable patients to refrain from engaging in unhelpful cognitive strategies as well as behaviors arising from their former metacognitive beliefs. However, to fully understand whether a mediator is specific to D-MCT, it is necessary to compare D-MCT mediators to those of another active treatment, for example, to a CBT group approach.

The current study has several strengths (RCT, blinded assessors, etc.), but naturally it is not without limitations. First, the HDRS mean score was 15.48 points, indicating that the patients had mild depression, and thus it is unclear whether the results also apply to more severely depressed patients. On the other hand, patients had experienced on average three depressive episodes and were hospitalized for the second time^10^, which may indicate that their current depressive symptoms might have underplayed the general severity of their disorder. Moreover, scores of the assessed MCQ subscales were largely comparable to those of other, more severely depressed patients^28–30^.

Second, we did not assess the inter-rater reliability of clinical interviews in the present study (MINI, HDRS). Because all patients were also pre-diagnosed by a clinician, this may have mainly affected HDRS scores. However, because each patient was assessed by the same rater over all three assessment points, the presented results should not be affected. Third, to verify the proposed causal relationship, more intermediate assessments (e.g., session by session) would be desirable in future studies, as well as the manipulation of potential mediators. Fourth, changes in dysfunctional cognitive and metacognitive beliefs were only assessed by the use of self-report instruments, and these instruments, as discussed, did not cover the entire range of the D-MCT. As a result, and as suggested by the partial mediation, important mediators may still exist, so it would be desirable to cross-validate the results, for example, with other measures such as implicit tests^24^.

In summary, our findings suggest that the key mechanisms of change in D-MCT are metacognitive beliefs, particularly NFC. As such, the current study provides further support for the importance of addressing metacognitive beliefs in the treatment of depression.
